# The use of individual and collective selection, optimisation and compensation (SOC) strategies and their association with work ability among senior workers

**DOI:** 10.1007/s10433-024-00821-1

**Published:** 2024-09-18

**Authors:** Annette Meng, E. Sundstrup, Lars Louis Andersen

**Affiliations:** National Research Centre for the Working Environment, Lersø Parkallé 105, 2100 Copenhagen, Denmark

**Keywords:** SOC model, SeniorWorkingLife, Job demands and resources, Occupational psychology, Sustainable workplaces

## Abstract

*Background* Selection, optimisation, and compensation (SOC) can be important strategies for maintaining work ability as we age. This study aimed to explore differences in self-reported individual and collective use of SOC strategies across job functions, as well as their association with self-rated work ability. *Methods:* In the third wave of the SeniorWorkingLife study, 10,798 workers aged 50 + , across the job function categories “Office work”, “work with people”, and “work in the field of production”, replied to questions about collective and individual SOC strategies and work ability. Using multiple regression, we modelled associations between SOC and work ability. *Results:* Associations between SOC and work ability were generally weaker among participants working in the field of production. Both individual and collective use of selection had much weaker associations with work ability in the job functions “office work” and “working with people”. In the job function “working in the field of production”, only collective compensation was positively associated with work ability while individual selection was significantly but negatively associated with work ability. *Conclusions*: The use of SOC may be particularly beneficial for older employees working with people. Optimisation and compensation may be the most important SOC strategies for maintaining the work ability of older employees working with people and doing office work. For older employees working in the field of production, collective optimisation may support the maintenance of work ability while reduced work ability may be associated with the use of individual selection as a “coping strategy”.

## Introduction

The need to prolong the working life is becoming increasingly salient because of the ageing population, potentially leading to a shortage of qualified labour and an economic strain on society. A measure to prolong working life in many Western countries includes increasing the retirement age (Eurofound 2021), however, this measure cannot stand alone. Societies and workplaces must ensure that older employees are able to and motivated to continue working until or beyond the official retirement age.

Reduced work ability is associated with the intention to leave the job or profession and risk of disability, and early retirement (Camerino et al. 2006; Fisher et al. 2016). Thus, if we want to prolong the working life of the labour force, we need to apply measures that help maintain the work ability of the employees.

The balance between job demands and resources is central to the individual's work ability (Ilmarinen et al. 1997). Ageing have been found to be associated with a decline in work ability, particularly after the age of 50 (Ilmarinen et al. 1997), and to be associated with a reduction in some resources such as physical function (Kamper et al. 2021; Newton et al. 2002) and some cognitive functions (Salthouse 2012, 2009). Therefore, measures that help older employees balance out job demands and resources can be expected to contribute to improving or maintaining their work ability.

**Fig. 1:**
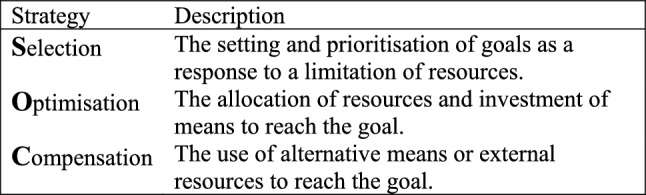
The SOC model adapted from Baltes and Baltes (1990)

The use of selection, optimisation, and compensation strategies (SOC) (Baltes and Baltes 1990) (Fig. 1) may help employees reach a better balance between job demands and resources. The SOC model was developed in the research field of successful ageing but has also been applied to the context of work (Moghimi et al. 2017). The SOC model identifies three types of action strategies that individuals use to manage limited resources. *Selection* is the setting and prioritisation of goals as a response to limited resources, i.e. we make choices about which goals are most relevant and meaningful to pursue in a given situation. *Optimisation* is the allocation of resources and investment of means to reach the goal, i.e. we make the most out of our resources to achieve the best possible outcome. *Compensation* is the use of alternative means or external resources to reach the goal (Baltes and Baltes 1990). The use of SOC strategies has been found to be positively associated with work ability (Becker et al. 2017; Müller et al. 2012a, b; Riedel et al. 2015; von Bonsdorff et al. 2014; Weber et al. 2018; Žmauc et al. 2019) supporting the idea that the use of SOC strategies contribute to a better balance between individual job demands and resources, and thereby improve or maintain work ability. The majority of studies on the association between the use of SOC strategies and work ability have been conducted on nurses. Although one study found that the association between the use of SOC and work ability was stronger among younger nurses (von Bonsdorff et al. 2014), the association has also been found to be stronger among older nurses (Müller et al. 2012a, b) and yet another study, including nurses aged 50 years or older, likewise reported a positive association (Žmauc et al. 2019). Studies including nurses of all ages also found positive associations between the use of SOC and work ability (Meng et al. 2022; Müller et al. 2012a, b; Müller et al. 2012a, b). These findings indicate that the use of SOC strategies may indeed support the work ability of older nurses.

When looking at research including other job groups, the results are more mixed. One study including health care professionals more broadly, including all ages, failed to find a significant association between the use of SOC and work ability (Weigl et al. 2013). Two studies analysing data from The LidA Cohort that include workers across job sectors found a positive association between the use of SOC strategies and work ability (Riedel et al. 2015) and only for the compensation subscale (Weber et al. 2018), respectively. Lastly, Sottimano et al. (2019) found a positive association between the use of SOC strategies and work ability in a sample of administrative workers of all ages. Although research on the use of SOC strategies has been conducted across various job groups, to the authors’ knowledge, no studies have explored differences between job groups in which categories of SOC strategies (selection, optimisation, compensation) are most commonly used. Nor have studies directly compared the strength of the association between the use of SOC strategies and work ability between job groups. Such knowledge may provide indications on which SOC strategies may be most relevant to address in interventions aiming to improve or maintain the work ability of older employees in different job groups.

Furthermore, researchers have recommended to explore the use of SOC strategies broader than the individual’s use of SOC strategies (Baltes and Dickson 2001; Baltes and Carstensen 1999; Moghimi et al. 2019; Müller et al. 2015). It has been argued that when using SOC strategies collectively, members of the team can contribute to defining goals (selection), in providing improved means (optimisation), and in offering alternative means when the individuals’ fail (compensation), potentially leading to higher levels of functioning for all members of the team (Baltes and Carstensen 1999). Often there is a high reliance on teamwork and cooperation between colleagues when solving the work tasks, making the collective use of SOC strategies particularly salient in a work context. Recently, research exploring the collective use of SOC strategies has emerged (Karlsen et al. 2022; Meng et al. 2021, 2022) and results indicate that the collective use of SOC strategies is associated with the work ability of employees (Meng et al. 2022). However, that study only included nurses. Therefore, more research exploring the association between the collective use of SOC strategies and work ability across different job functions is warranted.

The aim of the study was to explore differences in self-reported individual and collective use of SOC strategies across job functions (i.e. “Office work”, “work with people”, and “work in the field of production”), and to explore whether the job functions differ in the strength of associations between the self-reported individual and collective use of SOC strategies and self-rated work ability.

In the study, individual use of SOC refers to the participants’ personal use of SOC strategies at work. This could, for example, be to distinguish between “need to do” and “nice to do” tasks and decide to only do the “need to do” tasks on a busy day. While collective SOC use refers to the participants’ perception of their team’s collective use of SOC strategies, for example, that they collectively in the team prioritise between tasks on a busy day.

The study contributes with a first step in identifying job groups for whom the use of SOC strategies may be of greater importance in regard to the maintenance of the work ability of older employees. As well as indications of which SOC strategies may be more relevant to address in interventions to enhance or maintain the work ability of older employees in different job groups.

## Methods

### Design and participants

To explore the self-reported individual and collective use of SOC strategies and their association with self-rated work ability, we applied a cross sectional design analysing data from the third wave of the SeniorWorkingLife study.

The SeniorWorkingLife study is registered in ClinicalTrials.gov (Identifier: NCT03634410) as a cohort study and the protocol is published as open-access (Andersen and Sundstrup 2019). We employed data from the third wave of the study that was collected in medio 2022. In the third wave of the SeniorWorkingLife study, 10,798 workers aged 50 + , in the job function categories “office work”, “work with people”, and “work in the field of production”, replied to questions about collective and individual SOC strategies and work ability.

The following question was used to categorise the participants into job functions: “What do you work with first and foremost in your daily work?” with the following four response categories: (1) office work, administration, analysis, IT; (2) work with people, service, care; (3) work with processing, producing, or moving things, (4) other. In the analyses comparing the job functions, the participants were stratified into these response categories which were named (1) “office work”, (2) “work with people”, (3) “work in the field of production” (Andersen and Sundstrup 2019). Participants who had selected “other” as their job function were excluded from the analyses because they represent a mixed group with a very broad variety of job functions.

### Measures

#### Individual and collective use of SOC strategies

We used an adapted version of the questions to measure individual and collective use of SOC strategies developed by Meng et al. (2021). Collective use of SOC was measured with nine items, three representing selection strategies, three representing optimisation strategies, and three representing compensation strategies used in the team (see Table 2 for the items). Individual use of SOC strategies was likewise measured with nine items where each of the three categories of SOC strategies were represented by three items (see Table 2 for the items).

It was not possible to aggregate the results on the collective use of SOC strategies to the team-level or apply multilevel analysis, because we did not have entire teams or workplaces in our sample. Thus, we applied a self-perceived individual-level measure of both individual and collective use of SOC.

The questions were adapted to address the weaknesses pointed out by the authors (Meng et al. 2021). Furthermore, because the items had to be incorporated into the rather comprehensive questionnaire used in the SeniorWorkingLife study, it was necessary to reduce the response options from the five-point Likert scale originally used to a multiple choice format, where the participants were required to indicate whether they used each of the strategies presented rather than the extent to which they used them.

Thus, each participant could receive a score between 0 and 3 on selection, optimisation, and compensation, respectively, for both individual and collective use of SOC strategies. In addition, they could receive an overall SOC score between 0 and 9 for both individual and collective use of SOC strategies.

#### Work ability

Work ability was assessed with the question “How good do you think your current work ability is?” With response options on a scale ranging from 0 = “unable to work” to 10 “best work ability ever”, adapted from the Danish Psychosocial Work Environment Questionnaire (DPQ) (Clausen et al. 2019).

#### Control variables

Analyses were adjusted for age and gender from a national register, and for health and job demands from the questionnaire. Health was included because it is associated with work ability (Koolhaas et al. 2014; van den Berg et al. 2017) and the use of SOC strategies (Yeung and Fung 2009). Health was measured with the question “How do you think your health is overall?” with responses on a five point Likert scale from: 1 = excellent to 5 = poor, adapted from the Danish Psychosocial Work Environment Questionnaire (DPQ) (Clausen et al. 2019). Quantitative job demands are negatively associated with work ability (Riedel et al. 2015) and the use of SOC strategies (Abraham and Hanson 1995; Baltes and Heydens-Gahir 2003). Furthermore, high physical job demands are negatively associated with the work ability of employees with musculoskeletal pain (Skovlund et al. 2020). Job demands were measured with two questions adapted from the DPQ (Clausen et al. 2019): “How physically demanding do you find your current job?” and “How mentally demanding do you find your current job?” Both with responses on a scale rating from 0 = “Not strenuous at all” to 10 “Maximally strenuous”.

Job autonomy has been found to have a weak but positive association with the individual use of SOC (Müller et al. 2012a, b), and to interact with the associations between age, individual use of SOC and work ability (Weigl et al. 2013). Therefore, we tested for interactions between job autonomy and the respective SOC subscales in relation to work ability, but did not find any significant interactions when adjusting for multiple analyses (results are not shown but can be obtained by the authors on request). We therefore added job autonomy as a control variable in the regression analyses. Job autonomy was measured with the two questions “Do you have influence on when you perform your job tasks?” and “Do you have influence on how you perform your work tasks?” with response options on a five point Likert type scale ranging from 1 = always to 5 = never adapted from the DPQ (Clausen et al. 2019). In the analyses, the scales were normalised to a scale from 0 to 100 and the mean of the two scales calculated.

#### Statistics

Using multiple regression (Proc Reg, SAS version 9.4), we modelled associations between SOC strategies (explanatory variable) and work ability (outcome variable) stratified by job function category, reporting results as standardized beta coefficients. Analyses were adjusted for age, gender, health and job demands in the first models and further adjusted for job autonomy in the following models.

## Results

First, we present the descriptive results from the control variables and work ability, followed by the exploration of differences between the three job functions in the use of individual and collective SOC strategies. Thereafter, we present the results of the analyses of the strength of associations between the use of individual and collective SOC strategies and work ability, comparing the three job functions.

As shown in Table 1, a smaller proportion of participants working in the field of production were women, while women constituted the largest proportion of the participants working with people. Participants doing office work reported less physical job demands than the other job functions, while the three groups were quite similar regarding mental job demands. Participants doing office work reported higher levels of job autonomy than the other two job functions, who showed equal levels of job autonomy. Finally, a larger proportion of participants doing office work reported their health to be excellent or good and this group also reported the highest level of work ability (See Table 1).

**Table 1:** Descriptive results for control variables and work ability divided into the three job function categories

	Office work (*n* = 6721–7335)	Work with people (*n* = 436–2695)	Work in the field of production (*n* = 1822–2000)
Age (mean)	57	58	58
*Gender*			
Female (%)	47	67	16
Male (%)	53	33	85
*Job demands (mean (0–10)*			
Physical	1.4	4.3	5.7
Mental	5.0	5.9	4.8
*Health*			
Excellent or good health (%)	90	84	77
Job autonomy (mean 0–100)	79.8	71.4	70.9
Work ability (mean (0–10)	8.1	7.5	7.0

### Collective use of SOC

#### Collective selection

A smaller proportion of participants within the *field of production* reported to use of all three collective selection strategies, compared with the two other job functions. However, this difference was not statistically significant for the strategy “If we are under pressure, we jointly prioritise the work tasks in the group”. Furthermore, the smallest proportion of the participants in all three groups reported using the collective selection strategy to jointly find other points of time to perform some of the work tasks, when under pressure (see Table 2).

**Table 2:** Percentage of the participants who reported using each of the SOC strategies included in the questionnaire divided by job function category

	Percentage using the strategy		
	Office work (*n* = 6629)	Work with people (*n* = 2377)	Work in the field of production (*n* = 1792)
Collective use of SOC	% (95% CI)	% (95% CI)	% (95% CI)
*Selection*			
If we are under pressure, we jointly prioritise the work tasks in the group	46 (44–47)	46 (43–49)	40 (37–44)
If we are under pressure, we jointly find other points of time to perform some of the work tasks	27 (25–28)	28 (25–31)	**19** (17–22)
If someone in the group has difficulties in performing a work task, we exchange some of the work tasks	43 (41–45)	45 (42–49)	**35** (31–38)
*Optimisation*			
In my group, we usually help each other with heavy/demanding tasks	**35** (33–36)	40 (36–43)	**43** (39–46)
In my group, we encourage each other use ergonomically correct working postures	**14** (12–15)	26 (23–29)	28 (25–31)
In my group, we share new work-related knowledge with each other	58 (56–60)	52 (49–56)	**42** (39–45)
*Compensation*			
If we are under pressure, we ask to get help from someone from another team/department	29 (27–30)	31 (28–34)	30 (27–33)
If we have difficulties managing a work task in the group, we discuss if there are another way we can perform the task	42 (40–44)	40 (37–44)	**29** (26–32)
In my group, we usually encourage each other to use the technical assistive devices that are available to ensure safety and health	**18** (17–19)	32 (29–35)	36 (33–39)
Individual use of SOC	% (95% CI)	% (95% CI)	% (95% CI)
*Selection*			
If I feel under pressure, I deselect less important tasks	**47** (46–49)	**42** (39–45)	**31** (28–34)
If I feel under pressure, I postpone some of my work tasks to later	**63** (61–65)	**48** (45–51)	**36** (33–40)
If a work task puts too much strain on me, I ask to get removed from the task	**10** (9–11)	**17** (14–19)	11 (9–14)
*Optimisation*			
I usually make sure to use ergonomically correct working postures	**37** (35–38)	46 (43–49)	53 (49–56)
I take the breaks I need	50 (48–52)	**33** (30–36)	51 (48–54)
I put effort into learning new things that are important for my work	57 (55–58)	53 (50–56)	**42** (39–45)
*Compensation*			
If I have difficulties managing a work task, I ask my colleagues for help	47 (46–49)	50 (47–54)	52 (48–55)
If I have difficulties managing a work task, I try to find another way to perform it	40 (38–42)	40 (37–43)	41 (37–44)
I usually make use of the technical assistive devices available to ensure safety and health	**33** (31–34)	**44** (41–47)	**60** (57–63)

Significant differences are highlighted in bold. If only one value is highlighted it differs significantly from both of the other groups. If only two of the groups differ significantly from each other, the two relevant values are highlighted. If all three values are highlighted all three groups differ significantly from each other

#### Collective optimisation

The smallest proportion of the participants in all three job functions reported to encourage each other to use ergonomically correct working postures, and a significantly smaller proportion of participants doing *office work* than the two other groups reported to use this strategy. Furthermore, a significantly smaller proportion of participants *working in the field of production* than the other two groups reported sharing new working related knowledge with each other. Also, a significantly smaller proportion of participants doing *office work* than participants *working in the field of production*, reported that they usually help each other with heavy/demanding tasks in their group (see Table 2).

#### Collective compensation

A significantly smaller proportion of participants doing *office work* than the two other groups reported to encourage each other to use the technical assistive devices that are available to ensure safety and health. While a significantly smaller proportion of the participants *working in the field of production* than the two other job functions reported to discuss if there are other ways to perform the task if they have difficulties managing it (see Table 2).

### Individual use of SOC

#### Individual selection

All three groups differed significantly from each other in how large a proportion of the participants reported to postpone some of their work tasks to later if they feel under pressure and to deselect less important tasks when they feel under pressure, with the largest proportion being among participants doing *office work* followed by participants *working with people*, and the smallest proportion among participants *working in the field of production* in both cases. Furthermore, a significantly smaller proportion of participants doing *office work* than participants *working with people reported* to ask to get removed from a task if it puts too much strain on them. This was the individual selection strategy the smallest proportion of the participants in all three groups reported to use (see Table 2).

#### Individual optimisation

A significantly smaller proportion of participants *working in the field of production* than the other two job functions reported to put effort into learning new things that are important for their work. While a significantly smaller proportion of participants doing *office work* than the two other groups reported making sure to use ergonomically correct working postures. Furthermore, a significantly smaller proportion of participants *working with people* than the two other groups reported taking the breaks they needed (see Table 2).

#### Individual compensation

The largest proportion of participants *working in the field of production*, followed by participants *working with people*, reported to use the technical assistive devices available to ensure safety and health and these differences were all significant. There were no significant differences between the three groups in the proportion using the two other individual compensation strategies (see Table 2).

### The association between the use of SOC strategies and work ability

Results from Spearman’s R showed that all of the control variables (health, job demands, job autonomy) were significantly correlated with work ability. All SOC scales and SOC subscales except individual compensation were likewise significantly correlated with work ability. In addition, the SOC scales and subscales were mutually correlated. The correlation matrix is shown in Appendix 1. In the following, we present the results from the regression analyses.

#### Overall SOC scales

As shown in Table 3, collective use of SOC strategies had a stronger association with work ability than the individual use of SOC strategies across all three job functions. The associations between both individual and collective use of SOC strategies and work ability were strongest in the job function “*work with people*”, and weakest in the job function “*work in the field of production*”, where the associations were non-significant (See Table 3 Model 1).

**Table 3:** Results of regression for the two SOC scales with work ability as dependent variable

	Standardised beta		
	Office work (*n* = 5818–6606)	Work with people (*n* = 2087–2370)	Work in the field of production (*n* = 1487–1783)
*Model 1*			
Collective SOC	0.051***	0.097***	0.035
Individual SOC	0.041***	0.083***	0.009
*Model 2*			
Collective SOC	0.034**	0.077***	0.017
Individual SOC	0.033**	0.066***	-.006

Model 1 adjusted for gender, age, health, job demands. Model 2 additionally adjusted for job autonomy.. *P ≤ .05, **P < .01, ***P < .001

When additionally adjusting for job autonomy, the strength of association between the respective SOC scales and work ability were reduced in all three job groups and the level of statistical significance was reduced in the job function group “*office work*” (See Table 3 Model 2). None of the associations were statistically significant for the job function “*working in the field of production*”.

#### Subscales for collective use of SOC strategies

When looking at the strength of association between the subscales for collective SOC use and work ability, the job functions “*office work*” and “*working with people*” showed the same pattern. For both of these job groups, the collective use of optimisation followed by collective use of selection had the strongest association with work ability. However, all scales were significantly associated with work ability (See Model 3 in Table 4). When additionally adjusting for job autonomy, the strength of all associations was reduced, and the association between collective selection and work ability lost its statistical significance for the job function “*office work*” (See Model 4 in Table 4). The job function “*work in the field of production*” showed a different pattern of results than the other two job functions. In this job function category, collective compensation had the strongest association with work ability and collective optimisation was not significantly associated with work ability (See Model 3 in Table 4). When additionally adjusting for job autonomy, only collective compensation remained statistically significant associated with work ability (See Model 4 in Table 4).

**Table 4:** Results of regression for the subscales of collective SOC with work ability as dependent variable

	Standardised beta		
	Office work (*n* = 6606)	Work with people (*n* = 2370)	Work in the field of production (*n* = 1783)
*Model 3*			
Collective selection	0.029**	0.062***	0.046*
Collective optimisation	0.050***	0.086***	0.031
Collective compensation	0.047***	0.071***	0.051**
*Model 4*			
Collective selection	0.020	0.046**	0. 34
Collective optimisation	0.043***	0.071***	0.022
Collective compensation	0.040***	0.058***	0.042*

Model 3 adjusted for gender, age, health and job demands. Model 4 additionally adjusted for job autonomy. *P ≤ .05, **P < .01, ***P < .001

#### Subscales for individual use of SOC

When looking at the subscales for individual SOC use, the two job functions “*office work*” and “*working with people*” showed more or less the same pattern of results. Individual use of optimisation had the strongest association with work ability followed by individual compensation for both job functions and these associations were all statistically significant. Individual use of selection was not significantly associated with work ability for the job function “*office work*” (See model 5 in Table 5). When additionally adjusting for job autonomy, the strength of all associations was reduced and individual selection was no longer significantly associated with work ability for the job function “*working with peopl*e” either (See Model 6 in Table 5). Again the job function “*work in the field of production*” showed a different pattern of results. Only individual selection was significantly associated with work ability (See Model 5 in Table 5). When additionally adjusting for job autonomy, this association was strengthened and turned into a negative association (See Model 6 in Table 5).

**Table 5:** Results of regression for the subscales of individual SOC with work ability as dependent variable

	Standardised beta		
	Office work (*n* = 6606)	Work with people (*n* = 2370)	Work in the field of production (*n* = 1783)
*Model 5*			
Individual selection	− 0.009	0.036*	0.037 *
Individual optimisation	0.067***	0.092***	0.029
Individual compensation	0.026*	0.061***	0.021
*Model 6*			
Individual selection	− 0.012	0.022	− 0.052**
Individual optimisation	0.058***	0.073***	0.017
Individual compensation	0.024*	0.053**	0.012

Model 5 adjusted for gender, age, health and job demands. Model 6 additionally adjusted for job autonomy*P ≤ .05, **P < .01, ***P < .001

## Discussion

The aim of the study was to explore differences in self-reported collective and individual use of SOC strategies across the three job function categories: “office work”, “work with people”, and “work in the field of production”, and to explore whether the job functions differ in the strength of association between the self-reported individual and collective use of SOC strategies and self-rated work ability. Overall, the results showed that the extent to which the various SOC strategies were used by the participants within the three job functions, to a large extent reflected the nature of the work in the respective job functions. Furthermore, the association with work ability was stronger for the collective than the individual use of SOC strategies particularly among participants working with people, but none of the associations were significant for participants working in the field of production. Regarding the subscales, both individual and collective optimisation and compensations had the strongest associations with work ability in the job functions “office work” and “working with people”. While in the job function “working in the field of production”, collective compensation and individual selection had the strongest association with work ability, the latter being a negative association.

### Self-reported use of SOC strategies

When comparing the three job functions, the results show that a significantly smaller proportion of participants working in the field of production reported to use both the collective and individual selection strategies, which are strategies related to the exchange of work tasks between colleagues and when and whether to perform tasks as well as the collective optimisation strategy to find other ways to perform tasks. These strategies can be expected to require some level of job autonomy and job autonomy has been pointed out as a prerequisite for the use of selection strategies (Moghimi et al. 2017). Previous research indicate that employees working in the field of production have less control over when and whether to perform work tasks (Andersen et al. 2022). However, in our sample, this job function did not report lower levels of job autonomy than participants working with people. These results could indicate that the strategies are less salient for this job function and that they thus experience other barriers than lack of job autonomy to use them. To support the retention of older employees within the field of production, it may prove beneficial to explore if there are possibilities for enhancing the flexibility in who, when, and how to perform the working tasks.

A significant smaller proportion of participants working in the field of production reported to acquire and share work-related knowledge. This could reflect a greater focus on knowledge sharing and acquisition in the other two job functions that include knowledge workers and generally a larger proportion of employees with higher education levels. However, staying up-dated in the field and keeping up with the technological development at the workplace is important for all job functions to support the work ability and retention of senior employees (Müller et al. 2012a, b; Sundstrup et al. 2022). It may therefore prove beneficial for organisations within the field of production to have a greater focus on developing a culture, where employees are encouraged to and supported in the acquisition and sharing of work-related knowledge.

Furthermore, the results show that a significantly smaller proportion of the participants doing office work reported to use the optimisation strategies related to ensuring ergonomically correct use of the body as well as the compensation strategies related to the use of technical assistive devises. This could reflect the often higher levels of physical demands within the other two job functions, making the use of technical assistive devices more salient and perhaps reflecting a larger focus on its use. However, a significant smaller proportion of participants working with people than those working in the field of production reported to use technical assistive devices despite the fact that employees in healthcare and childcare also face high physical demands (Jakobsen et al. 2015; Rasmussen et al. 2018). These results could reflect that employees working in the field of production encounter fever barriers to using the assistive devises than, for example, employees in healthcare who are known to experience numerous barriers to use assistive devises (Andersen et al. 2019; Noble and Sweeney 2018).

A smaller proportion of participants working with people than the other two job functions reported to use the individual optimisation strategy “I take the breaks I need”. Breaks help employees maintain their energy levels at work (Sianoja et al. 2016) and therefore this optimisation strategy is important for the preservation of employee resources. Interventions addressing the insufficiency of breaks in this job function may thus support the employees in managing and preserving their resources, potentially supporting a prolonged working life among older employees.

Lastly, the proportion of participants working with people reporting to use the various SOC strategies often fall in between the two other job functions. Part of the explanation for this may be that this job function category probably presents the largest variation in job demands of the three categories, for example, teachers are not exposed to as high physical demands as health and personal care workers (Andersen et al. 2021). Furthermore, employees in many jobs in this category such as health and child care experience high both physical and mental job demands (Clipa and Boghean 2015; Mijakoski et al. 2018), and may thus need a broader range of SOC strategies to manage their resources.

### The association between Self-reported use of SOC strategies and self-rated work ability

The results show that the associations between both individual and collective use of SOC strategies and work ability were strongest for the job function “work with people”. These findings indicate that the use of SOC strategies may perhaps be particularly beneficial for the working ability of older employees working with people, and provide further support for previous research finding a positive association between the use of SOC and work ability (Meng et al. 2022; Müller et al. 2012a, b; Müller et al. 2012a, b; von Bonsdorff et al. 2014; Žmauc et al. 2019).

Across all three job functions, the collective use of SOC strategies had a stronger association with work ability than the individual use of SOC strategies. These associations were, however, not significant for the job function “working in the field of production”. Nevertheless, these findings may reflect the Nordic approach to organisational structure, where the employees are organised into teams that have high levels of influence on decisions and high participation in the organisation of the work tasks (Sørensen et al. 2012). This again may facilitate the use of collective SOC, because the employees have the freedom to organise their work in a way that allows them to take into account their strengths and weaknesses, benefitting the work ability of all members of the team (Baltes and Carstensen 1999). However, when adjusting for job autonomy, this trend was most evident in the job function “working with people”. These results support previous findings showing a stronger association between collective use of SOC strategies than individual use among nurses (Meng et al. 2022). It could be that this way of organising teams is more widespread in some job groups working with people, which could be part of the explanation for why the association between collective SOC use and work ability is stronger in this job function. Previous research has pointed out the challenges associated with addressing the individual use of SOC strategies in interventions (Müller et al. 2015). Together these findings point in the direction that interventions addressing the collective use of SOC strategies may be more beneficial, which is in line with the conclusion of a systematic review that interventions directed at the group rather than individual level are more effective at preventing accidents (Dyreborg et al. 2022), perhaps particularly in job groups that have greater interdependence and autonomy within teams when performing their work tasks.

### The association between the subscales of the collective use of SOC strategies and self-rated work ability

Among participants working with people, all three subscales remained significantly and positively associated with work ability when adjusting for job autonomy, with *optimisation* followed by *compensation* showing the strongest associations. These results support previous findings by Meng et al. (2022) who found that the collective use of *optimisation* and *compensation* had the strongest association with work ability in a sample of nurses. These findings could partly reflect that some job groups working with people are exposed to a broad range of job demands (Clipa and Boghean 2015; Mijakoski et al. 2018) making a broader range of SOC salient as well as the before mentioned diversity in job demands within this group of participants (Andersen et al. 2021), where the salience of the various SOC strategies are like to vary between the various job groups working with people. Nevertheless, the results indicate that all three types of collective SOC strategies potentially are important for the work ability of older employees working with people. However, because the data are cross-sectional, it is not possible to draw conclusions on the direction of the associations or causality (see also study limitations below). Future research comparing various job groups working with people and applying a longitudinal design allowing for conclusions on causality is encouraged to provide further nuances on which strategies may be particularly important for the work ability of older employees working with people.

Among participants doing office work, only collective *optimisation* and *compensation* remained significantly and positively associated with work ability when adjusting for job autonomy, and the associations were equally strong. The strategies, in these two SOC categories, the largest proportion of the participants reported to use were “to share new work-related knowledge” (optimisation) and “to discuss other ways to perform a work task if they had difficulties managing it” (compensation). It could be that this way of cooperating about the work tasks is particularly beneficial for the work ability of older employees doing office work. Again our cross-sectional design does not allow for conclusions on causality. It could also be reversed causality so that participants who experienced higher levels of work ability, due to their great work experience and knowledge, to a larger extent engaged in knowledge sharing and discussions on how to better perform difficult work tasks in their teams. If this is the case, it could perhaps support the work ability of their younger inexperienced colleagues benefiting the entire team (Baltes and Carstensen 1999).

Although the association between the overall collective SOC scale and work ability was low and not statistically significant in the job function “working in the field of production”, both *collective selection* and *compensation* were significantly and positively associated with work ability. However, when further adjusting for job autonomy, only *collective compensation* remained significantly associated with work ability. These findings indicate that the use of *collective optimisation* may not be important for the work ability of older employees in this job function. The *collective compensation* strategy the largest proportion of participants in this group reported to use was, to encourage each other to use technical assistive devises to support safety and health. It may be that this shared focus on using technical assistive devices is particularly beneficial for the work ability of older employees in this job function, often characterised by high physical demands.

### The association between the subscales of the individual use of SOC strategies and self-rated work ability

Participants doing office work and working with people showed the same pattern of results regarding the individual use of SOC. For both groups, *individual optimisation* and *compensation* had the strongest association with work ability and the association was strongest for *optimisation*. These results indicate that individual selection may be less important for the work ability of older employees doing office work and working with people. These results are in line with the findings of previous research on the individual use of SOC showing that only optimisation was associated with work ability in a sample of nurses (Meng et al. 2022), and results from a cohort study finding that only compensation had a positive but weak association with work ability (Weber et al. 2018).

The job function “work in the field of production” showed a different pattern of results compared to the other job functions. In this group, only *the individual use of selection* was significantly associated with work ability and when adjusting for job autonomy, this association turned negative. The fact that the association turned negative could indicate reverse causality where participants with lower work ability to a larger extent use individual selection strategies. It could be speculated that this could reflect that employees need to display reduced work ability to justify getting exempted from doing work tasks and thus, that selection is not used as a strategy to prevent loss of work ability of older employees. Individual compensation and optimisation was not significantly associated with work ability. This is somewhat surprising because they include strategies such as using technical assistive devises to ensure health and safety and to use ergonomically correct working postures, which one would expect to be particularly salient to balance out job demands and resources in this job function characterised by high physical job demands. Research using a longitudinal design is needed to provide insights into which strategies may be useful for preventing loss of work ability among older employees working in the field of production.

### Strengths and limitations

A major strength of this study is the large, representative sample of workers aged 50 years or older in Denmark. To counteract non-response bias common in questionnaire studies, all analyses were performed using statistical weights based on high-quality national registers ensuring that the estimates are representative of employees aged 50 years or older in Denmark (Andersen and Sundstrup 2019). Nevertheless, the results may not generalise to a younger population as older employees often have a greater need to find strategies to deal with declining work ability and perhaps benefit from different strategies than younger employees. Also, research in other national contexts is warranted to provide further support for the findings.

A limitation to the study is that the stratification of job functions into “office work”, “work with people”, and “work in the field of production” was based on a question about what the participants primarily worked with. A division into job functions based on register-based industry codes could have eliminated any bias associated with determining the job function based on self-reports. On the other hand, within the same industry, people can have different work functions, for example, in the construction industry there are both office work and manual work. Furthermore, a previous study applying the same categories found distinct differences between these three job functions (Sundstrup et al. 2022), supporting the validity of this categorisation.

A further limitation to the study is the cross-sectional design, which does not allow for conclusions about causal associations. Therefore, it cannot be ruled out that, for example, employees with higher levels of work ability have more resources to use SOC strategies. Intervention studies could help establish whether the associations found reflect a causal relationship where the use of SOC strategies actually affects the work ability of the employees. Nevertheless, because the purpose of the study was to compare the three job function categories, it is less of an issue in this study.

We applied a self-perceived individual-level measure of both individual and collective use of SOC. Research applying multi-level analyses of the collective SOC use is encouraged to achieve a stronger and more correct measure of the collective use of SOC in teams and its association with work ability.

Another limitation to the study is that all data included in the analyses were self-reported posing the risk of common method bias (Podsakoff et al. 2003), which may inflate the strength of the associations found.

The response options were reduced from the five-point Likert scale used in the original questionnaire into a multiple choice format. This is likely to have reduced the variance in the data because employees are likely to show variation in the extent to which they use the SOC strategies rather than just whether they use them or not. This may have led to an underestimation of the strength of associations found and may have blurred differences between the job function categories.

## Conclusion

The results indicate that overall, the use of both collective and individual SOC strategies is strongest associated with work ability among older employees working with people, followed by those doing office work, while the associations generally were notably weaker among those working in the field of production. These findings indicate that the use of SOC may be particularly beneficial among older employees working with people. Furthermore, results showed that for the two job functions “working with people” and “office work” particularly optimisation but also compensation both used individually and collectively were associated with work ability, while individual and collective selection had much weaker associations with work ability. These findings indicate that optimisation and compensation may be the most important SOC strategies to address when aiming to maintain the work ability of older employees working with people and doing office work. For participants working in the field of production, only collective selection was positively associated with work ability, while individual selection was negatively associated with work ability. These findings could indicate that while the use of collective optimisation strategies may support the maintenance of work ability, reduced work ability may be associated with the use of individual selection as a “coping strategy” rather than being used as a preventative measure to maintain work ability among older employees in this job function. Nevertheless, research applying longitudinal designs allowing for conclusions on causality is needed to shed further light on which SOC strategies are the most beneficial to address in interventions aiming to maintain the work ability of older employees across different job functions.

Lastly, the extent to which the specific SOC strategies were used by the three job functions, to a large extent reflected the nature of the work in the respective job functions. Nevertheless, differences in the barriers they encounter to use the SOC strategies may also influence the use of the strategies. Thus, research into barriers encountered to the use of SOC strategies and how to overcome these may provide important knowledge when designing interventions to enhance the work ability of the employees.

## Data Availability

The authors encourage collaboration and use of the data by other researchers. Data is stored on the server of Statistics Denmark, and researchers interested in using the data for scientific purposes should contact the project leader Prof. Lars Louis Andersen, lla@nfa.dk.

## Appendix 1

See Table

**Table 6:** Spearman’s R for the study variables

	1	2	3	4	5	6	7	8	9	10	11	12	13	14	15
1. WA	–	–	–	–	–	–	–	–	–	–	–	–	–	–	–
2. Age	− 0.12***	–	–	–	–	–	–	–	–	–	–	–	–	–	–
3. Gender	0.02*	− 0.09***	–	–	–	–	–	–	–	–	–	–	–	–	–
4. Health	0.55***	0.01	− 0.03**	–	–	–	–	–	–	–	–	–	–	–	–
5. P. JD	− 0.31***	0.02*	− 0.05***	− 0.25***	–	–	–	–	–	–	–	–	–	–	–
6. M. JD	− 0.11***	− 0.12***	− 0.01	− 0.15***	0.14***	–	–	–	–	–	–	–	–	–	–
7. JA	− 0.24***	0.02*	− 0.09***	0.18***	− 0.20***	− 0.17***	–	–	–	–	–	–	–	–	–
8. C. SOC	0.07***	− 0.03**	− 0.01	0.06***	0.02	− 0.05***	0.13***	–	–	–	–	–	–	–	–
9. I. SOC	0.08***	− 0.06***	− 0.02	0.07***	− 0.02*	− 0.01	0.08***	0.59***	–	–	–	–	–	–	–
10. CS	0.08***	− 0.04***	− 0.02	0.07***	− 0.04***	− 0.01	0.09***	0.79***	0.40***	–	–	–	–	–	–
11. CO	0.06***	− 0.05***	− 0.02	0.05***	0.02*	− 0.00	0.05***	0.82***	0.48***	0.55***	–	–	–	–	–
12. CC	0.05***	− 0.06***	0.02*	0.03**	0.04***	− 0.00	0.04***	0.81***	0.50***	0.53***	0.61***	–	–	–	–
13. IS	0.04***	− 0.07***	0.00	0.03**	− 0.12***	0.11***	0.05***	0.32***	0.62***	0.29***	0.22***	0.26***	–	–	–
14. IO	0.13***	− 0.01	− 0.04***	0.11***	− 0.04***	− 0.10***	0.13***	0.44***	0.80***	0.27***	0.38***	0.35***	0.24***	–	–
15. IC	0.01	− 0.05***	0.00	0.00	0.10***	− 0.02	− 0.00	0.55***	0.80***	0.36***	0.46***	0.51***	0.28***	0.49***	–

^*^significant at the *P* < 0.05 level, **Significant at the *P* < 0.01 level, ***significant at the *P* < 0.001

*WA* Work ability, *P. JD* Physical job demands, *M. JD* Mental job demands, *JA* Job autonomy, *C. SOC* Collective SOC, *I. SOC* Individual SOC, *CS* Collective selection, *CO* Collective optimisation, *CC* Collective compensation, *IS* Individual selection, *IO* Individual optimisation, *IC* Individual compensation. *n* = 9371–10,772

^6^.
